# Interpreting central hemodynamics in durable biventricular circulatory support patients

**DOI:** 10.1016/j.jhlto.2026.100554

**Published:** 2026-04-02

**Authors:** Xiaoman Xiao, Misha Dagan, Silvana Marasco, Hitesh C. Patel, Shane Nanayakkara, Justin Mariani, David McGiffin, David M. Kaye

**Affiliations:** a Department of Cardiology, Alfred Hospital, Melbourne, Australia; b Monash Alfred Baker Centre for Cardiovascular Research, Monash University, Melbourne, Australia; c Department of Cardiothoracic Surgery, Alfred Hospital, Melbourne, Australia

**Keywords:** Ventricular assist devices, Biventricular support, Hemodynamics, Pulmonary artery pressure, Blood flow, Mathematical modelling

## Abstract

**Introduction:**

Progressive developments in mechanical circulatory support devices have seen the increasing use of devices which directly augment pulmonary blood flow, including the deployment of durable pumps in the biventricular configuration (BiVAD). This paradigm introduces complex haemodynamic interactions which have not been fully characterized, compared to LVAD alone. We sought to investigate the effect of BiVAD support on central hemodynamics with particular regard to assessing pulmonary flow and vascular properties.

**Methods:**

This retrospective cohort study included LVAD and BiVAD patients who had right heart catheterization (RHC) performed within 6 months prior to MCS implantation and thereafter. Traditional RHC parameters, native LV and RV stroke volume (SV), and measures of pulmonary vascular function, including pulmonary arterial compliance (PAC) and elastance (E_PA_), were compared between BiVAD and LVAD patients.

**Results:**

A total of 62 patients, 13 (21%) BiVAD and 49 (79%) LVAD patients, were studied. At follow-up, despite similar baseline values, the BiVAD vs LVAD cohort had significantly higher pulmonary artery pressures (mmHg): sPAP (37 ± 14 vs 28 ± 11, *p* = 0.02) and dPAP (22 ± 8 vs 12 ± 6, *p* < 0.001). A reduction in PVR following BiVAD insertion was observed but remained significantly greater in BiVAD vs LVAD patients (2.1 ± 1.1 vs 1.6 ± 0.6 Wood Units, *p* = 0.02). Pulmonary arterial compliance (PAC), calculated using "total" stroke volume (SV) comprising the pulsatile and continuous flow components of the BiVAD configuration) was higher in BiVAD vs LVAD patients. However, using the native right ventricular SV, PAC was lower in the BiVAD cohort and remained unchanged compared to baseline. Simulation modeling demonstrated BiVAD support modestly increases pulmonary artery pressure and reduces right heart output, which was observed in our study.

**Conclusion:**

The presence of combined pulsatile and continuous flow in BiVAD patients significantly influences the hemodynamic profile compared to LVAD patients, particularly in regard to pulmonary pressures. Our study highlights the potential challenges in applying conventional pulmonary vascular hemodynamic indices in patients with continuous flow components.

## Background

Over the past decade, there has been a progressive increase in the deployment of continuous-flow (CF) ventricular assist devices (VAD) in a biventricular configuration (BiVAD) to support patients with significant concomitant right and left heart failure.^1^, ^2^, ^3^ The increase in BiVAD utilization likely reflects a range of factors, including increasing technical experience and improved hemocompatibility, together with the persisting challenge of late transplant referral.^4^ While CF-BiVAD with magnetic levitation (Mag-Lev) technology has improved survival compared to historical (and non-Mag-Lev) BiVADs,^1^ survival in BiVAD recipients remain significantly lower than that of LVAD recipients.^1^, ^5^

Severe pulmonary hypertension with elevated pulmonary vascular resistance in this cohort is common and is a contraindication to cardiac transplantation. Therefore, in addition to the augmentation of forward cardiac output, an added role of LVAD support is the reduction in left atrial pressure with consequent favorable effects on pulmonary arterial pressure and pulmonary vascular resistance. In patients requiring biventricular support, the hemodynamic interaction is far more complex, comprising a requirement to achieve adequate systemic blood flow whilst eliminating pulmonary congestion.^2^, ^6^, ^7^ Whilst addition of a right ventricular assist device (RVAD) affords adequate left ventricular filling, it is also accompanied by theoretically adverse effects of increased pulmonary blood flow resulting from the combination of RVAD output together with residual pulsatile right ventricular output. Current conventional measures of pulmonary vascular resistance and compliance do not take into consideration these complex interactions.

Despite these concerns, the influence of short- and intermediate-term BiVAD support on pulmonary arterial pressures and vascular resistance have not been investigated in detail. In addition, a description of the hemodynamic profile of BiVAD patients per se, consideration of appropriate means to best characterize pulmonary vascular function in the context of complex pulmonary blood flow is also important in regard to the assessment of readiness for heart transplantation. In particular, parameters including pulmonary effective arterial elastance (E_PA_) and pulmonary vascular resistance (PVR) are well established as risk factors for early graft dysfunction in LVAD patients undergoing transplant, this has not been definitively explored in BiVAD patients.^8^, ^9^, ^10^

Accordingly, we sought to characterize the effect of BiVAD support on central hemodynamics and to contrast them with observed patterns in LVAD patients and to explore the most appropriate means of assessing pulmonary vascular properties in BiVAD-supported patients.

## Methods

### Study design

We retrospectively reviewed the electronic medical records of all patients (*n* = 62) who underwent Heartmate 3 VAD implantation at the Alfred Hospital in Melbourne, Australia, during the period January 2016 to April 2024. Of this cohort, 49 patients underwent isolated LVAD implantation, whilst 13 patients received durable implantation of an LVAD and an RVAD (i.e., BiVAD). Patients were included if right heart catheter data was available within 6 months prior to VAD implant and thereafter on at least 1 occasion. Demographic information, echocardiographic, and past medical history was also collected. In 5 patients an initial strategy of temporary RV support was adopted, with conversion to durable RVAD due to failure to wean from temporary support at a median of 13 days (interquartile range: 12-15). In the remaining 8 patients, pre-operative assessment by the multidisciplinary team identified a high probability of need for durable RV support at the time of LVAD implant. The ultimate decision to implant durable left and right ventricular support at the same operation was made by the implanting surgeon intraoperatively, based on further visual assessment of the severity of the right ventricular dysfunction.

Right heart catheter studies were conducted in the non-fasted state using a 7 Fr balloon thermodilution catheter inserted under fluoroscopy via a right internal jugular venous sheath. Parameters recorded including right atrial (RAP), systolic, diastolic, and mean pulmonary arterial pressure (s/d/mPAP), and pulmonary capillary wedge pressure (PCWP). Cardiac output (CO) was performed via thermodilution in triplicate. In LVAD/BiVAD patients we also estimated left ventricular stroke volume as the difference between CO and estimated LVAD flow, and similarly for BiVAD patients, the right ventricular stroke volume was estimated as the difference between CO and RVAD flow. PVR was calculated conventionally as the ratio of the transpulmonary gradient (TPG) to the CO.

To explore pulmonary vascular properties in further detail, we also calculated the estimated pulmonary artery compliance (PAC) and pulmonary arterial elastance (Ea). These parameters are conventionally calculated as the ratio of the stroke volume to pulmonary artery pulse pressure and as the ratio of the pulmonary artery systolic pressure to stroke volume, respectively. In BiVAD patients we also calculated these parameters using the RV stroke volume, which accounts for the PA pulse pressure.

The study was approved by the Alfred Health Ethics Committee (project no. 221/24).

### Statistical analysis and computer modeling

Categorical variables are presented as frequencies and percentages. Means and standard deviation are described for continuous variables. Median and interquartile range are shown for categorical variables. Between group testing was performed using the Student *t* test for continuous parametric data, the Mann-Whitney test was used for non-parametric data and the $x^{2}$ test for categorical data. All tests were 2-tailed and assessed at the 5% significance level. Data analysis was performed using Statistical Package for the Social Sciences for Windows (SPSS version 29, IBM). All *p* values <0.05 were considered significant.

To further evaluate the "immediate" effects of mechanical circulatory support in the setting of advanced heart failure, we applied a computer modeling approach using the hemodynamic simulator Harvi.^11^ In this analysis, we simulated the effect of HM3 support in the LVAD configuration at a representative pump speed of 5,200 rpm alone and in conjunction with RVAD support at a pump speed of 4,000 rpm. The baseline HF physiologic profile was established using the average baseline pre-MCS hemodynamic profile of the study cohort.

## Results

Baseline characteristics for the total 62 patients are summarized in Table 1. There were 13 (21%) patients who underwent BiVAD implantation and 49 (79%) who underwent LVAD implantation. The BiVAD cohort (*n* = 13) tended to be younger than the LVAD cohort (*n* = 49) at the time of implantation (46 ± 15 vs 53 ± 10 years, *p* = 0.06). There was a high prevalence of non-ischemic cardiomyopathy in both cohorts (BiVAD vs LVAD: 92% vs 71%, *p* = 0.12). The duration between baseline hemodynamic assessment and VAD implantation was similar between LVAD and BiVAD patients: 0.4 ± 0.1 vs 0.5 ± 0.1 months. The time between VAD implant and follow-up assessment was longer in BiVAD vs LVAD patients (8.8 ± 6.5 vs 4.7 ± 1.9 months, <0.001).

**Table 1:** Patient Demographics

	BiVAD (*n* = 13)	LVAD (*n* = 49)	*p*-value
Age at VAD implant (years)	46 ± 15	53 ± 10	0.06
Male sex	13 (100%)	45 (92%)	0.37
Ischemic dilated cardiomyopathy	1 (8%)	14 (29%)	0.12
Non-ischemic dilated cardiomyopathy	12 (92%)	35 (71%)	0.12
Anthracycline	2 (15%)	2 (4%)	
Sarcoid	1 (8%)	0	
Arrhythmogenic cardiomyopathy	1 (8%)	0	
Past medical history			
Atrial fibrillation	7 (54%)	23 (47%)	0.66
Ischemic heart disease	1 (8%)	15 (31%)	0.10
Hypertension	2 (15%)	7 (14%)	0.92
Dyslipidaemia	3 (23%)	11 (22%)	0.96
COPD	0	1 (2%)	0.61
Chronic kidney disease ≥ stage 4	0	3 (6%)	0.37
Diabetes mellitus	5 (38%)	11 (22%)	0.25
Cerebrovascular accident (stroke, transient ischemic attack)	1 (8%)	5 (10%)	0.79

Abbreviations: BMI, body mass index; COPD, chronic obstructive pulmonary disease; RHC, right heart catheterization; VAD, ventricular assist device.

Comparative hemodynamic parameters prior to and post implantation of the LVAD or BiVAD are detailed in Table 2. Prior to VAD implantation, hemodynamic parameters between cohorts were similar, with each having elevated left and right-sided filling pressures, low cardiac index (CI), and elevated PVR. Post implantation, the BiVAD cohort had significantly higher pulmonary artery pressures: sPAP (37 ± 14 vs 28 ± 11 mmHg, *p* = 0.02), dPAP (22 ± 8 vs 12 ± 6 mmHg, *p* < 0.001) and mPAP (28 ± 9 vs 19 ± 8 mmHg, *p* = 0.002). PCWP was also marginally higher in the BiVAD group; however, was not statistically different (14 ± 6 vs 11 ± 6 mmHg, *p* = 0.07). Consequently, TPG (13 ± 6 vs 8 ± 3 mmHg, *p* < 0.001) and PVR (2.1 ± 1.1 vs 1.6 ± 0.6 WU, *p* = 0.02) were higher in the post-BiVAD cohort. The post-BiVAD cohort also had a higher CI (3.2 ± 0.6 vs 2.8 ± 0.6 L/min/m^2^, *p* = 0.046) than the post-LVAD cohort. RAP:PCWP was lower in the post-BiVAD than the post-LVAD cohort (0.4 ± 0.3 vs 0.7 ± 0.4, *p* = 0.01).

**Table 2:** Comparative Hemodynamic Parameters at Baseline and During HM 3 BiVAD or LVAD Support

	Pre-BiVAD (*n* = 13)	Pre-LVAD (*n* = 49)	*p*-value	Post-BiVAD (*n* = 13)	Post-LVAD (*n* = 49)	*p*-value
RAP (mmHg)	11 ± 7	10 ± 6	0.74	6 ± 4	7 ± 4	0.37
sPAP (mmHg)	52 ± 16	58 ± 15	0.21	37 ± 14	28 ± 11	0.02
dPAP (mmHg)	26 ± 6	29 ± 7	0.23	22 ± 8	12 ± 6	<0.001
mPAP (mmHg)	37 ± 9	40 ± 9	0.29	28 ± 9	19 ± 8	0.002
PCWP (mmHg)	24 ± 5	27 ± 7	0.27	14 ± 6	11 ± 6	0.07
CI (L/min/m^2^)	1.9 ± 0.4	1.9 ± 0.6	0.98	3.2 ± 0.6	2.8 ± 0.6	0.05
CO_TD_ (L/min)	3.8 ± 0.9	3.9 ± 1.1	0.92	6.3 ± 1.0	5.5 ± 1.1	0.03
Fick CO (L/min)	3.4 ± 1.2	3.5 ± 1.2	0.92	5.7 ± 1.2	5.1 ± 1.4	0.37
PVR (WU)	3.3 ± 1.9	3.6 ± 1.9	0.68	2.1 ± 1.1	1.6 ± 0.6	0.02
TPG (mmHg)	13 ± 7	13 ± 5	0.88	13 ± 6	8 ± 3	<0.001
RVSWI (g/m^2^/beat)	593 ± 316	694 ± 278	0.28	918 ± 407	428 ± 225	<0.001
RAP:PCWP	0.5 ± 0.3	0.4 ± 0.2	0.17	0.4 ± 0.3	0.7 ± 0.4	0.01
SV (mL)	46 ± 11	48 ± 17	0.74	88 ± 20	73 ± 16	0.006
PAC (mL/mmHg)†	2.4 ± 1.7	2.1 ± 1.6	0.56	7.5 ± 3.7	5.4 ± 3.1	0.06
Ea (mmHg/mL)‡	1.2 ± 0.5	1.3 ± 0.5	0.39	0.4 ± 0.2	0.4 ± 0.2	0.52
LVAD Flow (L/min)				5.2 ± 0.2	4.7 ± 0.1	0.007
RVAD Flow (L/min)				4.0 ± 1.3	-	

Data are mean ± SD. Abbreviations: CI, cardiac index; CO_TD_, cardiac output-thermodilution method; dPAP, diastolic pulmonary artery pressure; Ea, pulmonary arterial elastance; Fick CO, Fick cardiac output; mPAP, mean pulmonary artery pressure; PAC, estimated pulmonary arterial compliance; PCWP, pulmonary capillary wedge pressure; PVR, pulmonary vascular resistance; right RAP, right atrial pressure; RVSWI, right ventricular stroke work index; sPAP, systolic pulmonary artery pressure; SV, stroke volume; TPG, transpulmonary gradient; WU, Wood Unit.

Patients who required upfront durable RVAD (dRVAD) implantation had features of poorer RV function compared to those with initial temporary rVAD (tRVAD) support as reflected by their pre-implant hemodynamics: RA pressure (14 ± 8 vs 7 ± 3 mmHg, *p* < 0.05); PA systolic pressure (42 ± 11 vs 63 ± 14 mmHg, *p* < 0.05); PA mean pressure (33 ± 7 vs 43 ± 8 mmHg, *p* < 0.05); PCWP (24 ±;5 vs 25 ± 4 mmHg, p = ns); RV stroke work index (423 ± 325 vs 774 ± 207 mmHg.mL/m2, *p* < 0.05). Consistent with the hemodynamic observations, patients requiring durable BiVAD support had features of more advanced RV dysfunction compared to LVAD-only patients as reflected by their RV dysfunction score: 2.5 (2.3-3) vs 2 (1-2.4), *p* < 0.01.

**Figure 1:**
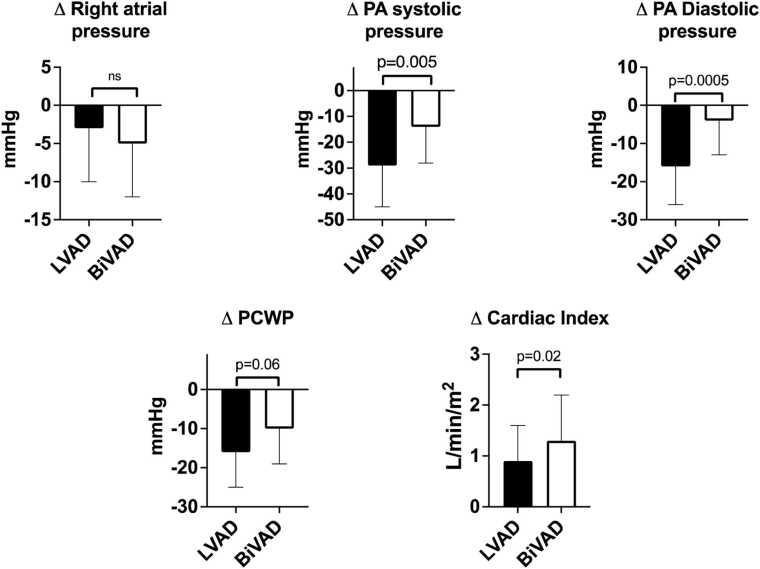
Bar graphs represent the change in hemodynamic parameters with LVAD and BiVAD support.

As shown in Figure 1, there were important differences in the changes in hemodynamics following implantation of LVAD compared with BiVAD support. A greater reduction in PA systolic pressure was demonstrated in the LVAD than the BiVAD group (−29 ± 16 vs −14 ± 14 mmHg, *p* = 0.006) and these differences were even more prominent for the PA diastolic pressure in the LVAD compared to the BiVAD group (−16 ± 10 vs −4 ± 9 mmHg, *p* = 0.0005). A greater reduction in PCWP was demonstrated in the LVAD than the BiVAD group (−16 ± 9 vs −10 ± 9 mmHg, *p* = 0.056) although this was not statistically significant. The increment in total CI was greater in BiVAD compared to LVAD patients (1.3 ± 0.4 vs 0.999 ± 0.7 L/min/m^2^, *p* = 0.22).

Despite the implantation of an RVAD in the RA to PA configuration, a pulsatile PA profile pressure was evident in all patients. The PA pulse pressure was similar in BiVAD compared to LVAD patients (15 ± 9 vs 16 ± 1 mmHg, *p* = 0.80). To estimate the PVR, we first applied the simple ratio of the TPG to the CO. As shown in Table 2, prior to the implantation of the VAD, the PVR was similar in BiVAD vs LVAD patients (3.3 ± 1.9 vs 3.6 ± 1.9 Wood Units). Following VAD, the PVR decreased but remained significantly greater in BiVAD patients (2.1 ± 1.1 vs 1.6 ± 0.6 Wood Units. *p* = 0.02). Given that the CI was greater in BiVAD patients, it was apparent that the higher PVR in BiVAD patients was a function of the greater TPG (13 ± 5 vs 8 ± 3 mmHg, *p* < 0.001). To further characterize the pulmonary circuit, we next applied other conventional measures, including calculation of the pulmonary arterial compliance (PAC) and pulmonary arterial elastance (E_PA_). The PAC and E_PA_ were similar at baseline. Following a VAD, the conventionally estimated PAC was higher in BiVAD vs LVAD (7.5 ± 3.7 vs 5.4 ± 3.1 mL/mmHg, *p* = 0.057), whilst E_PA_ remained similar (0.4 ± 0.2 vs 0.4 ± 0.2 mmHg/mL *p* = 0.52).

**Figure 2:**
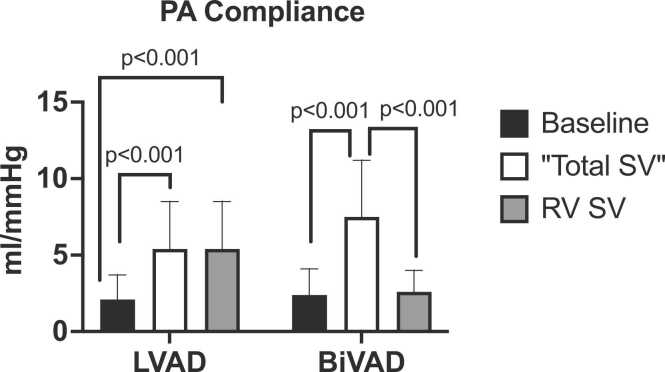
Bar graphs demonstrate the change in estimated pulmonary arterial compliance with LVAD and BiVAD support when calculated conventionally and with specific RV stroke volume.

Implantation of an RVAD converts the pulmonary circulation from a pulsatile circuit to a combined pulsatile and continuous flow, and therefore we considered that the conventional measures of pulmonary vascular function may not be applicable. We first estimated the native left and right ventricular stroke volume from the differences between estimated VAD flow and CO. The LV stroke volume tended to be greater in BiVAD vs LVAD (21 ± 10 vs 14 ± 9 mL *p* = 0.05) and with smaller residual RV stroke volume in BiVAD patients (32 ± 14 vs 73 ± 16 mL). We next used the residual RV stroke volume to estimate the PAC as shown in Figure 2. These data indicated that the PAC in BiVAD patients was consistent with that observed at baseline. Consistent with this approach, the ratio of PA systolic to native RV stroke volume (i.e., E_PA_) was greater in BiVAD patients given the smaller residual native stroke volume.

**Figure 3:**
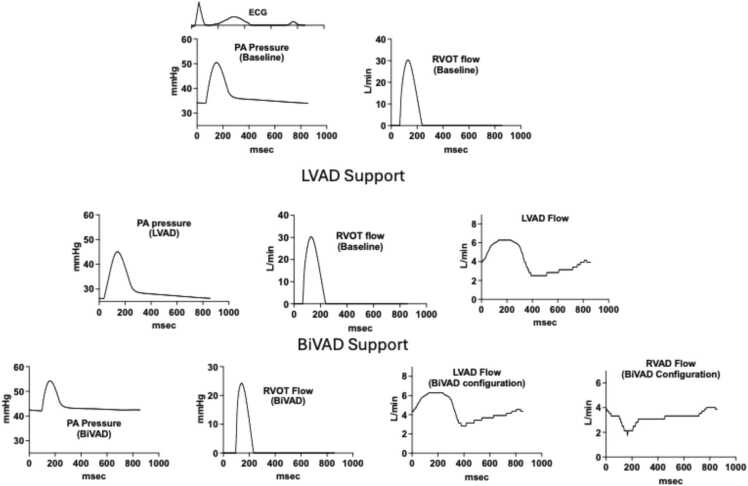
Graphs represent hemodynamic simulation of the effect of LVAD or BiVAD support.

Finally, to further understand how the implantation of an LVAD or BiVAD influences pulmonary artery pressure and right heart output, we performed a simulation using the mathematical model Harvi. As shown in Figure 3, RVAD support (in addition to an LVAD) is predicted to modestly increase pulmonary artery pressure and reduce right heart output, as seen in our clinical study. Interestingly, and in contrast to an LVAD, RVAD support in the RA to PA configuration is associated with a relative reduction in RVAD flow during RV ejection, and RA contraction is modeled to increase flow.

## Discussion

The objective of the current study was to detail and compare the hemodynamic profile of patients with biventricular versus left ventricular continuous flow mechanical circulatory support. We demonstrated that compared to LVAD patients, BiVAD patients have 1) higher pulmonary artery pressures, cardiac index, and PVR with 2) less overall reduction in pulmonary artery pressures and a trend towards lower reduction in PCWP.

In particular, a central objective of our study was to understand how implantation of a CF-RVAD impacts upon the pulmonary circulation and how routine assessment of PVR may be complicated by the conversion to a combination of pulsatile and continuous flow.^2^, ^7^ In a normal heart, pulsatile loading of the pulmonary circulation by the right ventricle is reflected in the sPAP.^11^, ^12^ In the current study, we documented the presence of pulsatile flow in the pulmonary artery of BiVAD patients, which is indicative of ongoing contractile function of the right heart sufficient to exceed the pulmonary artery pressure and thus to eject through the pulmonary valve.

Hemodynamic assessment represents an important component of the management of patients with durable MCS devices irrespective of the intent of the support. In particular, detailed physiologic measurement of native cardiac and total CO can assist in optimization of pump speed and in the evaluation of cardiac recovery or in the setting of hemodynamic compromise, for example, with aortic incompetence or tamponade. Measurement of filling pressures is appropriate where clinical uncertainty exists regarding volume state, valvular disease, or pericardial constraint. Finally, assessment of the PVR is critical in the case of potential transplant listing and in the setting of right heart failure. In the setting of biventricular MCS support, the thermodilution method and the Fick method can be applied to assess total right heart output right heart output.^13^ Specifically, given the configuration of the CF-RVAD with an inflow cannula in the RA, CO and thus SV indicates the combined RVAD and intrinsic RV output. A comprehensive measure of the RV load in the BiVAD configuration requires consideration of the pulsatile and resistive components, noting that the RV is significantly afterload sensitive.^12^, ^14^ PVR reflects the resistive component of the RV load on the pulmonary arterial vasculature.^15^ The reduction in PVR with LVAD implantation is well-described in literature, and a residual raised PVR may be associated with increased mortality post heart transplantation.^8^, ^16^, ^17^, ^18^ Pulmonary hypertension in the setting of left heart failure results from an elevated left ventricular filling pressure and resultant pulmonary vascular remodeling and increased pulmonary vascular tension, leading to a disproportionate increase in pulmonary artery pressures.^19^, ^20^, ^21^ The reduction in pulmonary artery pressure with LVAD implantation is achieved through off-loading the left ventricle with a resultant reduction in TPG and PVR.^18^

In the present study, we found that the PVR estimated conventionally from the TPG and the total CO yielded a significantly higher residual PVR in BiVAD patients. Given the implications of this finding, we explored whether other parameters of pulmonary vascular function might provide other insights. Both PAC and Ea incorporate resistive and pulsatile components of the RV load and have been shown to be predictors of mortality in left heart failure even when the resistive component (PVR) is normal.^12^, ^15^, ^22^, ^23^ Several studies have demonstrated the disproportionate reduction in PAC to PVR in patients with left heart failure and an elevated PCWP, thus increasing the RV pulsatile load.^12^, ^24^, ^25^ PAC has been demonstrated to be inversely related to PVR and PCWP, and reduction of left-sided filling pressures with medical therapy can improve PAC.^25^, ^26^ Further, a reduced PAC is associated with poor prognosis in patients with left-sided heart failure and right heart failure due to pulmonary arterial hypertension.^25^, ^27^, ^28^ E_PA_ has also been associated with increased mortality in patients with pulmonary hypertension due to left-sided heart failure and a strong predictor of severe right heart dysfunction following LVAD implantation.^10^, ^12^, ^14^ Although the PCWP was modestly higher in BiVAD patients, the difference was not significant compared to LVAD patients. Similarly, the RA pressures were not different, suggesting that the volume status of the two patient groups was broadly managed appropriately.

Using the lumped approach, our study estimated a rapid increase in PAC post-BiVAD insertion. Similarly, in LVAD patients, an increase in PAC was also observed as a function of increased stroke volume and lower pulse pressure. In LVAD patients an increase in compliance could theoretically result from a reduction in pulmonary vascular distension. Mathematically, the even greater increase in PAC in BiVAD patients is a product of the increased RV SV relative to LVAD patients alone through a combination of the pulsatile and continuous RV flow (despite pulse pressure being unintuitively similar). Though mechanistically, this greater increase in PAC in BiVAD patients is somewhat harder to resolve, and therefore we considered the pulsatile element which generated the pulmonary systolic pressure (i.e., RV ejection). In this calculation, the PAC was in fact unchanged from baseline in BiVAD patients; however, remained improved in LVAD patients. We also assessed for changes in E_PA_ between the BiVAD and LVAD cohorts. Use of the "total" stroke volume and PA systolic pressure did not demonstrate any differences; however, inclusion of the smaller RV ejection volume did lead to estimation of a higher elastance, potentially due to a more pressure-loaded pulmonary circulation. It is possible these observations may also reflect the fact that RV failure is often (depending on the pathology) a reflection of more advanced left heart disease with fixed pulmonary vascular remodeling and resultant RV-PA uncoupling. Therefore, upon removing continuous flow from the equation, the failure of PAC to improve post-implant may reflect fixed pathology. Regardless, these data highlight the important limitation of conventional assessments of compliance in the setting of mixed pulsatile and continuous flow.

A significant challenge in the management of RVAD patients is the ability to accurately evaluate RVAD flow and RV output independently. The HM3 flow estimator has not been validated in the RA to PA configuration to the best of our knowledge. Assessment of RV output, for example, via Doppler echocardiography, is frequently very challenging due to the presence of limited echocardiographic windows.

Our study has several potential limitations. As outlined above, the intent of the manuscript was to highlight the hemodynamic profile of patients with an RVAD (in this case BiVAD) and to contrast it with LVAD hemodynamics. We did not seek to compare the hemodynamic benefit per se, although parameters were similar at baseline. Similarly, our manuscript was not focused on hemodynamic prediction of the need for durable RVAD support. Indeed our experience (like most centers) is that, when required, temporary RV support can be discontinued in patients undergoing LVAD implantation. In this cohort all BiVAD patients underwent LVAD implant with temporary RVAD support, with the intent of RVAD weaning, but this was not possible due to insufficient native RV delivery to the LVAD. Although not the focus of this study, it highlights the need for further work in predicting RV function post-LVAD.

Firstly, this is a single-center study with a small patient cohort. Owing to the retrospective nature of this study, many VAD patients did not meet the inclusion criteria due to the absence of RHC performed within the required study timeframe were excluded from the study. Furthermore, this study had a predominance of male patients and a large population of patients with non-ischemic cardiomyopathy, which may reduce generalizability of the results to the female gender and patients with ischemic cardiomyopathy.

In conclusion, our study highlights important differences in the pulmonary hemodynamic profiles of patients with BiVADs compared to LVADs, principally because of the presence of combined pulsatile and continuous flow. In particular, our data raise concerns over the use of "conventional" hemodynamic indices of pulmonary vascular function when continuous flow is present and in their comparison with baseline pulsatile flow indices. Such a challenge may be even more relevant in the context of total artificial heart device deployment as a bridge to transplant in patients with elevated PVR. In contradistinction to BiVAD support of the native heart, total artificial heart devices deliver either entirely pulsatile flow (Syncardia) or continuous flow (eg, Bivacor), thereby delivering differing pulmonary artery blood flow and pressure dynamics. Furthermore, serial comparison of PVR is confounded by the inability to access the central pulmonary circulation. In this situation, assessment of pulmonary artery pressure via a previously placed pulmonary artery pressure sensor has been reported to be of potential utility.^29^ Our study is limited by small numbers. Taken together, the present study highlights the need for better measures of pulmonary vascular "resistance" to flow, which will likely require the real-time acquisition of continuous pressure and flow and where appropriate assessment of vasodilator responsiveness.

## Disclosures statement

DK has served on an advisory panel for Abbott.

## Conflicts of Interest statement

The authors declare the following financial interests/personal relationships, which may be considered as potential competing interests: David Kaye reports a relationship with Abbott that includes consulting or advisory. If there are other authors, they declare that they have no known competing financial interests or personal relationships that could have appeared to influence the work reported in this paper.
